# Pleural Solitary Fibrous Tumors—A Retrospective Study on 45 Patients

**DOI:** 10.3390/medicina56040185

**Published:** 2020-04-16

**Authors:** Cornel Savu, Alexandru Melinte, Radu Posea, Niculae Galie, Irina Balescu, Camelia Diaconu, Dragos Cretoiu, Simona Dima, Alexandru Filipescu, Cristian Balalau, Nicolae Bacalbasa

**Affiliations:** 1 Department of Thoracic Surgery, “Marius Nasta” Institute of Pneumonology, 050152 Bucharest, Romania; alexandru.melinte@gmail.ro (A.M.); radu.posea@gmail.ro (R.P.); niculae.galie@gmail.ro (N.G.); 2 Department of Thoracic Surgery, “Carol Davila” University of Medicine and Pharmacy, 020021 Bucharest, Romania; 3 Department of Surgery, “Ponderas” Academic Hospital, 021188 Bucharest, Romania; irina_balescu206@yahoo.com; 4 Department of Internal Medicine, “Floreasca” Clinical Emergency Hospital, 105402 Bucharest, Romania; drcameliadiaconu@gmail.com; 5 Department of Internal Medicine, “Carol Davila” University of Medicine and Pharmacy, 020021 Bucharest, Romania; 6 “Alessandrescu-Rusescu” National Institute of Mother and Child Health, Fetal Medicine Excellence Research Center, 020395 Bucharest, Romania; dragos.cretoiu@gmail.ro; 7 Department of Cell and Molecular Biology and Histology, “Carol Davila” University of Medicine and Pharmacy, 020021 Bucharest, Romania; 8 Center of Excellence in Translational Medicine, Fundeni Clinic Institute, 022328 Bucharest, Romania; simona.dima@gmail.ro (S.D.); nicolae_bacalbasa@yahoo.ro (N.B.); 9 Department of Obstetrics and Gynecology, “Carol Davila” University of Medicine and Pharmacy, 020021 Bucharest, Romania; alexandru.filipescu@gmail.ro; 10 Department of Obstetrics and Gynecology, “Elias” Emergency Hospital, 105402 Bucharest, Romania; 11 Department of Surgery, “Carol Davila” University of Medicine and Pharmacy, 020021 Bucharest, Romania; cristian.balalau@gmail.ro; 12 Department of Surgery, “Pantelimon” Clinical Hospital, 021661 Bucharest, Romania; 13 Department of Obstetrics and Gynecology, “I Cantacuzino” Clinical Hospital, 030167 Bucharest, Romania

**Keywords:** PSFT, resection, surgery

## Abstract

*Introduction:* The purpose of this paper is to study the type, the clinical presentation, and the best diagnostic methods for pleural solitary fibrous tumors (PSFTs), as well as to evaluate which is the most appropriate treatment, especially as PSFTs represent a rare occurrence in the thoracic pathology. *Material and Method:* A retrospective study was conducted on a group of 45 patients submitted to surgery between January 2015 and December 2019. In most cases, the diagnosis was established through imaging studies—thoracic computed tomography (CT) scan with or without contrast—but also using magnetic resonance imaging (MRI) or positron emission tomography (PET) scans when data from CT scans were scarce. All patients were submitted to surgery with curative intent. *Results*: Most patients included in this study were asymptomatic, with this pathology being more common in patients over 60 years of age, and more common in women. The occurrence of malignant PSFT in our study was 17.77% (8 cases). All cases were submitted to surgery with curative intent, with a single case developing further recurrence. In order to achieve complete resection en bloc resection of the tumor with the chest wall, resection was performed in two cases, while lower lobectomy, pneumectomy, and hemidiaphragm resection, respectively, were needed in each case. Postoperative mortality was null. *Conclusion*: Thoracic CT scan remains the most important imagistic investigation in diagnosing. MRI is superior to thoracic CT, especially in cases that involved the larger blood vessels within the thorax, spinal column, or diaphragm. Complete surgical resection is the gold standard in treatment of PSFT, and the prognosis in benign cases is very good.

## 1. Introduction

Pleural solitary fibrous tumors (PSFTs) are rare tumors, and their evolution is considered unpredictable. The incidence of this disease is considered to be lower than 5% of the total number of pleural tumors [1]. The first case of PSFT was described from a histological view by Wagner in 1870 [2], however, the first pleural tumor was presented by Lieutaud in 1767 [3,4]. In 1931, Klemperer and Rabin published a histopathological description and divided the tumors in two categories: diffuse and localised [5]. Stout and Murray (1942) were the first to identify the mesenchymal origin of pleural tumors, which was later confirmed by electronic microscopy and immunohistochemistry [6]. Over time, these tumors have had different names: localised mesothelioma, benign mesothelioma, fibrous mesothelioma, pleural fibroma, benign pleural fibroma, pleural fibromyxoma, localised fibrous tumor, and so on [7].

Over the last 20 years, the term mesothelioma was replaced by solitary fibrous pleural tumor. These tumors were first described as being within the pleura, but it was later observed that it can also have an extra pleural localisation. There were also cases described of fibrous solitary tumors in various locations: liver, pelvis, peritoneum, meningeal, adrenal gland, intrapulmonary, urinary bladder, pericardium, and almost every organ system [8,9].

PSFTs are found within the pleura in 57.7% of cases, with the rest (42.3%) being localized extrapleural [8,10]. The World Health Organization (2015) reviewed the classification of pleural tumors from a pathological view and placed them in three categories: mesothelial tumors, mesenchymal tumors, and lympho-proliferative disorder. Both benign and malignant solitary fibrous tumors are part of the mesenchymal tumor group, along with desmoid tumors and calcified fibrous tumors.

## 2. Materials and Methods

This paper represents a retrospective study on a series of 45 patients diagnosed with PSFT who were submitted to surgery in our thoracic surgery clinic from the Institute of Pneumology, Bucharest over a period of five years (2015–2019). After obtaining the approval of the Ethical Committee no 12/9 February 2020, data of these patients were retrospectively reviewed. The analyzed parameters were represented by age, sex, clinical presentation, blood test results, imaging aspects, histopathological examination of the tumor after surgery, type of surgical resection, complementary treatment, and postoperative evolution. For a better classification of our series, we used the tumor size criteria (larger or smaller than 10 cm), with the De Perrot staging of pleural fibrous tumors (Table 1).

Differentiation between malignant and benign PSFT was done using the criteria established by England et al. [11]: presence of tumoral necrosis; presence of atypical nuclei, cellular pleomorphism, and hypercellularity; presence ≥4 mitosis/10 HPF (high power field). Immunohistochemistry tests were performed for both types of PSFTs.

Imaging studies consisted of chest X-ray, thoracic computed tomography (CT) scan, magnetic resonance imaging (MRI), and positron emission tomography (PET) scan. Both CT guided biopsy as well as direct tumor biopsy were used for histological diagnosis. Bronchoscopy was used in larger tumors with compression of the lung with a visible impact on the bronchial tree. Other routine tests performed were electrocardiography (EKG), lung function tests, blood gas levels, and transthoracic echography associated with a cardiology exam. Patients were also classified according to their smoking status as well as according to their exposure to asbestos or ionizing radiation.

Follow-up was done with the following protocol: standard chest X-ray at three and six months postoperative, thoracic CT scan every six months in the first two years and once a year for the next five years. Most patients from our series are still in the postoperative follow-up program, as we have set a 15 year monitoring period.

## 3. Results

From our 45 patient series, 35 were women (77.7%) and were 10 men (22.2%) with a ratio of 1 male/3.5 female (1:3.5). The age of our patients was between 32 and 84 years with an average of 61.84 years. The representation of age groups was as follows: 1 case between 30 and 40 years old (woman) (2.22%), 5 cases between 40 and 50 years old (women) (11.1%), 6 cases between 50 and 60 years old (5 women, 1 male) (13.3%), 19 cases between 60 and 70 years old (14 women and 5 male) (42.22%), 11 cases between 70 and 80 years old (7 women and 4 male) (24.44%), and 3 cases over 80 years old (2 women and 1 male) (6.66%).

**Figure 1:**
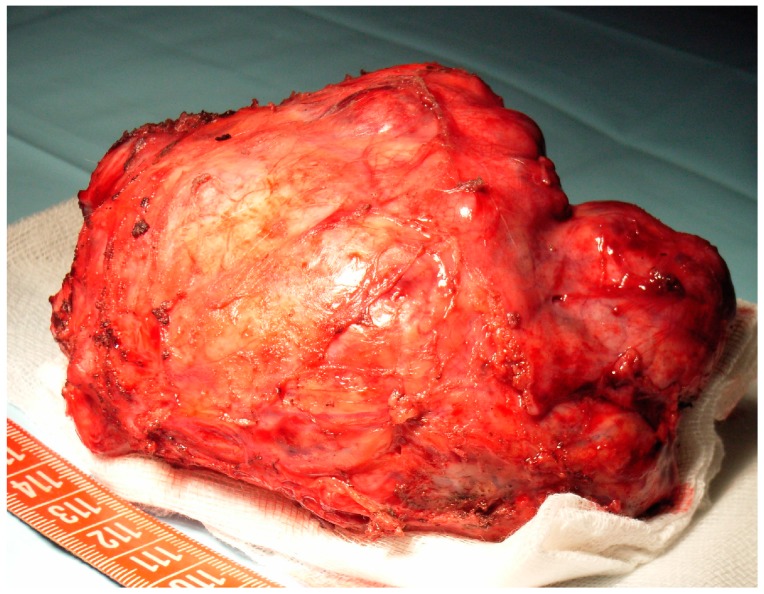
Surgical specimen.

According to the size of the tumors, in the case of PSFT, the largest was 34/24/15 cm and weighed 3800 g, and the smallest was 2.5/1.7/1.5 cm with a weight of 64 g. In our series, 60% of the tumors were under 10 cm (27 cases), and 40% over 10 cm (18 cases) (Figure 1).

Regarding the risk factors in our series, 15 cases (33.33%) were smokers, 2 cases were exposed to asbestos (4.44%), 1 case had a genetic factor (2.22%) (mother with pleural mesothelioma), 1 case had exposure to ionising radiation (2.22%), and 1 case had exposure to benzene (2.22%). In conclusion, 20 cases (44.44%) had exposure to risk factors.

From a clinical view, 24 cases (53.33%) were asymptomatic and 21 cases (46.66%) had a diversity of clinical manifestations (Table 2).

We can observe that most symptomatic presentations (14 cases—31.11%) were with chest pain, dyspnea, or cough. The rest of the symptoms found are associated with paraneoplastic syndrome: Doege–Potter syndrome, Pierre–Marie–Bamberger syndrome, arthralgia, articular oedema, or weight loss. Moreover, a correlation between the tumor size and symptoms was noted. Most asymptomatic patients had tumors <10 cm (23 cases—51.11%), with only one case presenting with chest pain (2.22%), with *p* < 0.01. From the symptomatic patients (21 cases—44.44%), 20 of them had tumors >10 cm with only one case (2.22%) under 10 cm. Regarding tumor localisation, 20 cases (44.44%) were in the right hemithorax and 25 cases (55.55%) in the left hemithorax. As a point of origin of the tumor, 21 cases (46.66%) where in the parietal pleura and 22 cases (48.88%) in the visceral pleural, with one case (2.22%) in the mediastinal pleural and one case (2.22%) in the left hemidiafragm.

Using de Perrot staging and England pathology criteria, there were 19 cases in stage 0, 18 cases in stage I, 1 case in stage II, 7 cases in stage III, and no cases for stage IV. Benign tumors (82.22%) were discovered in stages 0 and I, while malignant tumors (17.77%) were diagnosed in stages II and III (Table 3).

**Figure 2:**
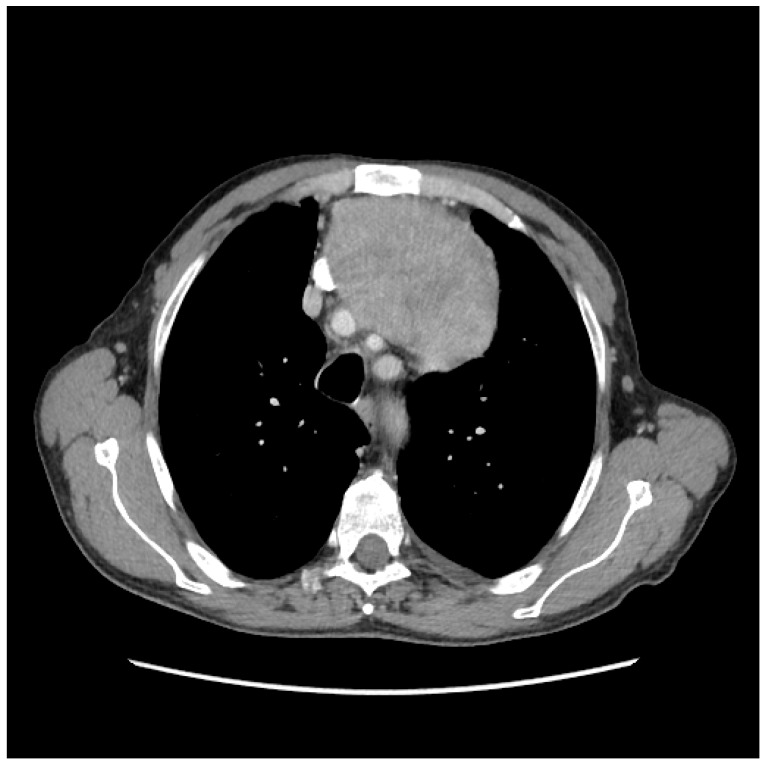
Computed tomography (CT) aspect.

Immunohistochemistry studies were used in 15 cases (33.33%) for both histological types. These tests were positive for cluster of differentiation 34 (CD34), B cell lymphoma (bcl-2), Vimentin, cluster of differentiation 99 (CD99), and signal transducer and activator of transcription 6 (STAT 6) in eight cases of malignant PSFT (17.77%), and were negative in seven cases (15.55%) of benign PSFT. Imaging diagnosis was based on simple chest X-ray, which was performed for all 45 patients. In 39 cases (86.66%), nodular or pleural masses were identified, two cases (4.44%) presented a normal aspect, while the remaining four cases (8.88%) were thought to have pulmonary or mediastinal masses. Further on, CT scan was performed in 35 cases (77.77%), of which 15 patients (42.85%) were diagnosed with pleural fibrous tumors, pleural mesothelioma was suspected (14.28%) in 5 cases, while a clear diagnosis could not be set in 8 cases (22.85%) (Figure 2). In another four cases (11.42%), a mediastinal tumor was suspected, while in three cases (8.57%), benign pulmonary tumors were suspected.

In six cases (17.14%), further investigations were performed, consisting of biopsy through thoracotomy in three cases (8.57%) and CT guided biopsy in another three cases (8.57%). In four cases (8.88%), along with thoracic CT scan, an MRI was performed in order to establish a diagnosis. In total, nine patients received an MRI (20%), in cases in which we suspected spinal involvement (two cases—4.44%), mediastinal blood vessels involvement (six cases—13.33%), or diaphragmatic invasion (one case—2.22%). Only one case in which a malignant thoracic tumor was suspected was submitted to a PET scan.

Comparing patients investigated through thoracic CT scan (35 cases) with those who received an MRI (nine cases), we noticed a higher diagnostic accuracy in cases in which MRI was used. Diagnosis was established by CT scan in 42.85% of cases (15 patients), while MRI established a clear diagnosis in all nine patients (100%). This further proves the greater accuracy of MRI studies when compared with thoracic CT scan in cases in which spinal column, blood vessels, or diaphragmatic invasion is suspected.

Treatment of PSFT consisted of surgery in all 45 cases, with only one case (2.22%) having a recurrence that required another surgical procedure in association with chemotherapy and radiotherapy.

En bloc surgical resection with 2 cm margins surrounding the tumor was performed in 38 cases (84.4%). The tumor was resected en bloc with chest wall resection (involving the first three ribs) in one case (2.22%), lower left lobectomy in one case (2.22%), left pneumonectomy in one case (2.22%), partial resection of the left hemidiaphragm in one case (2.22%), and posterior chest wall resection (involving the third, fourth, and fifth ribs) in one case (2.22%).

The correlation between the type of PSFT and the type of performed surgical procedure is presented in Table 4. Open surgery was performed in most cases (40 cases—88.8%) and video assisted thoracic surgery (VATS) surgery only in five cases (11.11%).

Most cases reported no postoperative complications. However, there were eight cases (17.77%) that required an extended postoperative stay, the most commonly encountered postoperative complications consisting of bleeding in two cases (4.44%), upper gastrointestinal bleeding owing to a gastric ulceration in one case (2.22%), cardiac arrhythmias in one case (2.22%), surgical wound infection in one case (2.22%), and paralysis of the left hemidiaphragm in one case (2.22%). The overall mortality was null.

Follow-up was structured in two intervals: an initial period for the first five years, during which 36 patients were introduced (80% of cases), with the other nine cases (20%) having yet to come in. The second period for follow-up will be until 15 years postoperative. Follow-up PET-CT scan was performed for only one patient with malignant PSFT, who after five years presented with local recurrence. PET-CT was not usually performed in order to differentiate between malignant or benign PSFT.

Two-year follow-up from surgery reviewed 34 cases (75.55%) and five-year follow-up reviewed 7 patients (15.55%). Of those reviewed after two years, 6 patients had malignant PSFT (with malignant histologic characteristics) (75%) and 28 patients had benign PSFT (benign histological characters) (62.22%). At the five-year follow-up, we only have five cases of benign PSFT and two cases of malignant PSFT. We mention that the five-year follow-up on all 45 patients is not yet completed.

## 4. Discussion

PSFTs are not as common as other types of pleural tumors, such as diffuse malignant pleural mesothelioma. They account for less than 5% of cases of pleural tumors and are more frequently encountered in men, during the sixth or seventh decade of their life [12]. Other authors consider it more common in women, with a proportion of 1:1.35 [11]. In our observation, we encountered a higher preponderance of women compared with men (77.7% of cases), with the proportion of women to men being 3.5:1.

Even though this affection may occur at any age, the sixth decade of life indicates a higher prevalence, as mentioned in the literature [13,14]. In our study group, we encountered an average age of 61.84, with the majority of cases being situated in the 60–70 age group (19 cases, or 42.22%) and in the 70–80 age group (11 cases, or 24.44%), similar to the data from the literature.

In the literature, there is no suggestive mentioning of the factors that may lead to PSFT development, excepting one published case, which mentioned the genetic character of this disease [15]. In addition, exposure to asbestos, tobacco, or other nitrogen oxide gases was not linked to PSFT. In our series, even though there were patients with previous exposure to smoking, asbestos, ionizing radiation, or benzene (19 cases—42.22%), a correlation could not have been established between exposure and PSFT development.

Some authors consider that up to 87% of cases diagnosed with PSFT have their origin in the visceral pleura and only 13% of them in the parietal pleura [16]. However, in our cases, a heterogenic localization was found, with an even 22 cases (48.88%) in the visceral pleura and 21 cases (46.66%) in the parietal pleura. As for the cellular origin of PSFT, the opinions are very diverse, leading to the numerous names of this disease. At present, the origin is accepted to be a mesenchymal one, at the level of the mesenchymal cells of the pleura. Nonetheless, there were cases of fibrous solitaire tumors with extrapleural localization at the level of the urinary bladder [17], retroperitoneum [18], salivary gland, meninges, thyroid, paranasal sinuses, or even with intrapulmonary location.

**Figure 3:**
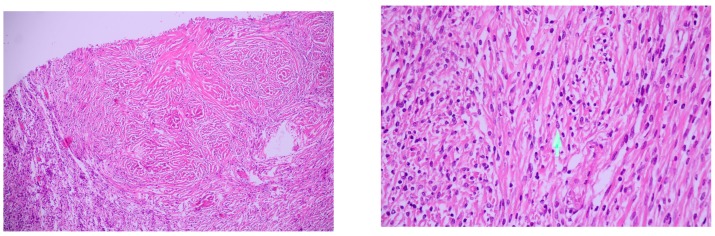
Histopathological aspects.

PSFT is a benign tumor in most cases, but in some cases, it can have malignant characters. The proportion of benign PSFT and malign PSFT is 7:1 [19]. The incidence of malign PSFT is considered as 12% of the entire cases of solitary fibrous tumors [20]. In our study, the majority of cases were benign (82.22%), and only eight cases were malign PSFT (17.77%), which is a higher number than those presented in the literature (Figure 3).

Some others consider that the incidence of malign PSFT is much higher, with the percentile reaching 43% of the cases [21]. Even though, it is accepted that approximately 78–88% of the cases represent benign PSFT and only 12% to 22% represent malignant cases [22]. In this context, there were some major criteria of malignancy put in place: mitotic index higher than 4/10 HPF, intratumoral necrosis or haemorrhage, pleomorphic cells, and the presence of metastasis; a minor criterion is represented by a size larger than 10 cm [13].

The histopathological diagnosis is usually accompanied by immunohistochemistry studies. The cases of malign PSFT are usually positive for CD35, CD99, bcl-2, or vimentin [23]. In our case, immunohistochemistry was used in 15 cases (33%), representing a disadvantage in getting a rigorous, definitive diagnosis. In addition, for a complete diagnosis, the Ki-67 antigen, associated with cellular proliferation, should be used (MIB-1-monoclonal antibody) for a better histological differentiation of the two cases of PSFT. Ki-67 is a nuclear protein, used as a marker of cellular proliferation, and MIB-1- monoclonal antibody for proliferation, linked with Ki-67. A Ki67 level <1% is suggestive for the lack of malignancy [1]. A more biological aggressiveness of PSFT is characterised by higher values of Ki-67 and p53 and variable CD-34 levels [7].

The recent oncological studies have shown, in the cases of PSFT, a fusion of NAB2–STAT6 gene, which is a result of intrachromosomal inversion 12q13.3., characteristic for this type of disease. This type of fusion leads to the development of STAT6 antibody, an immunohistochemical marker for this type of tumor [24]. Moreover, the mitotic number equal to higher than 4/10 HPF, TERD promoter, and mutation TP53 were correlated with the biological aggressiveness of the tumor [25]. NAB2–R593W fusion plays a major role in malign PSFT. Meanwhile, FLT1–R593W and KDR–V2971I somatic mutations are proof of the existence of a malignant angiogenetic phenotype [10].

There are correlation studies between sanguine inflammatory parameters and types of PSFT, which is the reason that fibrinogen, C reactive protein, and the neutrophil to lymphocyte ratio >5 were measured. An increase in these parameters was observed, especially in tumors larger than 10 cm, and especially in advanced stages from De Perrot classification as markers of malignity [26].

PSFTs are in most cases asymptomatic, but with their growth, they can lead to compression of the nearby organs (lungs, heart, large vessels, and thoracic wall), associated with a diversity of clinical symptoms [9,27,28]. In our case, the proportion of symptomatic and asymptomatic patients was almost equal, with 21 of them (46.66%) being symptomatic and 24 of them (53.33%) being asymptomatic. It should also be mentioned that, in most cases, the symptomatology was associated with the presence of larger tumors, with the size of over 10 cm—in our study, we have noticed that the symptomatology in patients with tumors larger than 10 cm was present in 44.44% of the cases, and in only one patient (2.22%), with a tumor smaller than 10 cm (who presented chest pain due to thoracic wall compression). The most frequent paraneoplastic syndromes mentioned in the literature are Doege–Potter syndrome (hypoglycaemic syndrome), hypertrophic pulmonary osteoarthopathy (Pierre–Marie–Bamberger syndrome), or the growth of the levels of beta human chorionic gonadotropin (BHCG) [29]. Refractory hypoglycaemia in PSFT context was first described in 1930, independently, by Doege and Potter, with no particularities in men or women incidences being observed [30,31,32]. Actually, hypoglycaemia associated with PSFT is considered a non-islet cell tumor hypoglycaemia (NICTH). NICTH is associated with solitary fibroma, hemangiopericytoma, or myxofibrosarcoma [33].

NITCH is encountered in approximately 4–5% of PSFT cases [34], and can be present in solitary pleural fibroma as well as in pelvic ones [35,36]. In our study group, out of 45 cases, Doege–Potter syndrome was present in two patients with malignant PSFT (4.44%). It is worth mentioning that NICHT can be associated with PSFT, regardless of the localization, but is more frequently associated with the malignant form (60.3%), representing an important prognostic factor [23,34]. In our study, out of the malignant forms of PSFT (eight cases), Doege–Potter was encountered in 25% of cases (six cases). Some studies have shown that PSFTs associated with hypoglycaemia represent 68% of the cases of pediculate tumors [19]. In our study group, we remarked that PSFTs were sessile or inverted tumors with histological sign of malignity. The main reasons for NICTH are the following: high level glucose usage by the tumor cells; insulin receptors proliferation; high levels of insulin like growth factor (IGF)-II, owing to tumor secretion; as well as a decrease in peptide C insulin, gonadotropin hormone (GH), and IGF-I. To sum up, the most important aspect of this pathology is the high level of IGF-II, as well as a significant change in IGF-II/IGF-I proportions (>10), and can confirm the NICTH diagnosis [37,38].

NICTH syndrome disappears with the tumor resection, a few days after the surgical procedure (3–4 days) [35]. In the cases of tumors that cannot be surgically removed, presenting with hypoglycaemia, some authors suggest alternative treatments such as a combination of two agents (Temozolomide and Bevacizumab), or radio embolization with yttrium 90 (Y^90^)-labelled glass microsphere [36,37]. Some other authors consider that, even if the tumor was completely removed, an adjuvant oncologic treatment is still needed [39]. Moreover, for an intraoperative control of glycaemia, it is recommended to use an artificial pancreas, which is capable to continually monitor glycaemia, and permanently adjust glucose and insulin intake [40].

Another paraneoplastic syndrome associated with PSFT is hypertrophic pulmonary osteoarthopathy, or Pierre–Marie–Bamberger syndrome [41]. In our study, the incidence was 4.44% (two cases) of malign PSFT. Causes of clubbing of the fingers and toes and of hypertrophic pulmonary osteoarthopathy are as follows: an abnormal production of hepatocyte growth factor (HGF) or a high level of hyaluronic acid, secreted by the fibrous tumor [16]. Digital clubbing can be measured using Schamroth sign, indicating the severity of the digital modification. However, they disappear with the complete tumor removal in two to six months from intervention [42]. We have acknowledged this with our two cases that presented Pierre–Marie–Bamberger syndrome—digital clubbing disappeared in about 2–5 months post-operative. Superior vena cava syndrome associated with PSFT is rarely described, even though it is frequently associated with bronchopulmonary cancer or lymphoproliferative disorders [43]. It is more frequently associated with malign PSFT [44]. In our cases, this syndrome was encountered in only one patient (2.22%) and was associated with malign PSFT, which leads to the apparition of arm and cephalic extremity oedema owing to the compression of superior vena cava and of the right atrium.

From a radiological view, the most frequent diagnosis is achieved through a simple thoracic radiography, in posterior–anterior incidence, which can determine the diagnosis of thoracic disease, as we have used in our research. Most commonly, the diagnosis is then confirmed using computed tomography of the thorax. For a complete diagnosis, with a definitive demarcation between malign PSFT and benign PSFT, a multislice computed tomography is used. This method can create a multidimensional reconstruction, revealing the exact localization and size (less or more than 10 cm), tumor density, and vascularization, as well as the existence of pleural effusion, which can be a criteria of malignancy of PSFT [45]. Usually, in thoracic CT scans, in PSFT cases, there are some criteria that should be studied: the connection between the tumor and the mediastinal pleura (acute or obtuse angle formed with the adjacent pleura), tumor characteristics (lobulated, smooth with clear edges), shape of the tumor, homogenous or heterogeneous aspects, and associated characteristics. These are represented by the presence or absence of lymphadenopathy and pleural metastasis, or of the cleavage plane between the tumor and mediastinal structure, the “geographic pattern” of a rich vascularization of the tumor, and the necrosis or calcifications. All these aspects of the CT scan can reveal the differences between malign and benign PSFT [46,47].

In contrast, some authors consider these aspects to not be definitive for a proper demarcation between malignant and benign PSFT, underlining the fact that there are no clear differences that could be revealed by CT scan [48]. Nonetheless, computed tomography was used as a first-line investigation in our case, being the elective investigation in 35 of the cases (77.77%). The accuracy of the diagnosis was 42.85% (15 cases). Later, in 20% of the cases (nine cases), the investigation was then followed up with thoracic MRI. It had the patenting to supply with quantitative and qualitative data, as well as anatomical and functional ones without using radiation from the administeration of nephrotoxic contrast dye, making it excellent to be used in differential diagnosis of malign or benign pleural diseases [49]. In addition, it is useful in determining the existence of mediastinal invasion (larger vessels, main bronchi, and trachea), of the spinal cord or of the diaphragm, conferring precious information for the surgical treatment. The postoperative histopathological diagnosis enriches the accuracy of the CT diagnosis, as was emphasized in three cases (6.66%) using cutting needle biopsy with CT guidance [49]. In three other cases (6.66%) of large tumors, adjacent to the thoracic wall and approachable by standard radiology guiding, we also used direct needle biopsy. We consider these types of procedures (fine needle biopsy of the tumor) as not mandatory, as they do not influence the necessity of surgical removal. Still, they are useful in cases in which the tumor cannot be excised or metastases are present.

The elective treatment in PSFT is surgical resection. In malignant cases, an en bloc extensive and complex resection is proposed (thoracic wall, pulmonary lobe, lung, diaphragm, and so on), using oncological margins. The survival rate in malignant PSFT is considered by some authors to be 68% [9]. Malignant PSFTs metastasize using the hematogenous path to the liver, central nervous system, spleen, bones, kidneys, and adrenal glands [9]. In our study, out of eight cases of malignant PSFT, just one case had a recurrence two years after the first surgical intervention, and needed another intervention associated with adjuvant oncological treatment.

According to some authors, even benign PSFT can be unpredictable (10–15% of cases), especially if the size of the tumor is larger than 10 cm and has infiltrated the pleural margins [7]. This is the reason why in the case of our patients, tumor resection was done with oncological margins of 2 cm and in association with histopathological examination from pleural margins (37 cases, 82.22%). Some other authors stated that cancer recurrence in benign PSFT is between 11.2% and 20%, with a prolonged follow-up being required for this reason [27,50]. Some other authors expressed that, in the case of recurrence of benign PSFT, surgical treatment is the main option, eith oncologic treatment remaining as the sole option for inoperative tumors or in the presence of metastatic disease [20,51].

Even though surgical treatment is essential, some other authors consider that in the cases of malignant PSFT, adjuvant oncologic treatment should be associated [9]. From our eight patients with malignant PSFT, we have considered the surgical treatment to be the key treatment of this pathology. In our opinion, adjuvant oncologic treatment is reserved for recurrent cases (one case, 2.22% from our study). The prognosis of patients with PSFT is good, with the majority of cases being benign (88%), and only 12% showing a malignant behaviour [14]. Some other authors consider the recurrence to be 16% in the cases of malignant PSFT and only 2% in the benign cases [21]. In our study, we have observed that malignant recurrence represents 12.5% of the cases (n = 1). This represented the recurrence at about two years after the first surgery, and now, it is one-year cancer free since the moment of reintervention and the end of adjuvant oncologic treatment. For benign PSFT, there was no disease recurrence. It is also worth mentioning that the follow-up of the 45 patients is not completed yet. There were 34 patients who were re-evaluated two years after surgery (75.55%), with seven of them being also re-evaluated at the five-year follow-up (15.55%). As the follow-up period is not yet finished for the whole study group, it is premature to conclude, as we have proposed a 15-year follow-up period.

Tapias proposes an evaluation score and late follow-up of the patients, based on a series of pathologic signs: the origin of the tumor in parietal pleura, sesil morphology of the tumor, size larger than 10 cm, hypercellularity, high necrosis, and mitotic activity. On the basis of this score, a recurrence free survival interval of 100% was determined, if the score is less than three points [31].

Tumor recurrence is the main risk factor that can lead to death, appearing mostly in malign PSFT, even with en bloc resection of the multimodal treatment and even with close follow-up [21].

## 5. Conclusions

In conclusion, PSFTs are rare pleural tumors, most of which are benign; moreover, most of them are associated with a good prognostic, with the rates of malignant lesions being under 20% of cases. Most often, they are asymptomatic, with no signs and symptoms for a long period of time, and being usually discovered at a routine chest X-ray. In our study, PSFT was most commonly found in women over 40 years of age. Most of the cases were in patients over the age of 60 years. Thoracic CT scan is the main radiological instrument used for diagnosing this disease. MRI is complementary and, in some cases, superior to a CT scan. As for the therapeutic strategy, en bloc surgical resection with negative oncological margins is the gold standard in the treatment of PSFT. Chemotherapy and radiotherapy should be used in recurrent disease or inoperable stages.

## Figures and Tables

**Table 1:** Pleural solitary fibrous tumor (PSFT) staging De Perrot.

Stage 0	Pedunculated Tumor Without Signs of Malignity
Stage I	Sessile or “inverted” tumor without signs of malignity
Stage II	Pedunculated tumor with histological signs of malignity
Stage III	Sessile or “inverted” tumor with histological signs of malignity
Stage IV	Multiple synchronous metastatic tumors

**Table 2:** Clinical presentation in PSFT.

Symptoms	Number of Cases
Hypertrophic osteoarthropathy Pierre–Marie–Bamberger syndrome	2 (4.44%)
Hypoglycemia Doege–Potter Syndrome	2 (4.44%)
Thoracic pain	5 (11.11%)
Cough	6 (13.33%)
Dyspnea	3 (6.66%)
Facial and upper body oedema Superior vena cava syndrome	1 (2.22%)
Arthralgia and articular oedema	1 (2.22%)
Weight loss	1 (2.22%)

**Table 3:** Malignant PSFT—correlation between symptoms, tumor size, and staging (De Perrot).

Symptoms	Size	Stage
Symptoms of Doege–Potter	34 cm	III
Doege–Potter syndrome	21 cm	III
Pierre–Marie–Bamberger syndrome	23 cm	III
Pierre–Marie–Bamberger syndrome	25 cm	III
Superior vena cava syndrome	15 cm	III
Arthralgia and articular oedema	18 cm	III
Weight loss	9 cm	II
Dyspnea	24 cm	III

**Table 4:** Surgery performed according to the histopahological form of PSFT.

Malignant Tumors	Benign PSFT
Tumoral resection (2.22%)	Tumoral resection 37 cases (82.22%)
Tumoral resection en bloc with left pneumonectomy (2.22%)	
Tumoral resection en bloc with left chest wall resection involving the first three ribs (2.22%)	
Tumoral resection en bloc with lower left lobectomy (2.22%)	
Tumoral resection en bloc with upper right lobectomy (2.22%)	
Tumoral resection en bloc with right chest wall resection involving the third, fourth, and fifth ribs (2.22%)	
Tumoral resection en bloc with left pneumonectomy (2.22%)	
Tumoral resection en bloc with partial diaphragm resection (2.22%)	

## References

[1] Yagyu H., Hara Y., Murohashi K., Ishikawa Y., Isaka T., Woo T., Kaneko T. (2019). Giant Solitary Fibrous Tumor of Pleura Presenting Both Benign and Malignant Features. Am. J. Case Rep..

[2] Wagner E. (1870). Das tuberkelahnliche Lymphadenom (Der cytogene oder reticulirte Tuberkel). Arch. Heilk. (Leipzig).

[3] Kucuksu N., Thomas W., Ezdinli E.Z. (1976). Chemotherapy of Malignant Diffuse Mesothelioma. Cancer.

[4] Ehrenhaft J.L., Sensenig D.M., Lawrence M.S. (1960). Mesotheliomas of the Pleura. J. Thorac. Cardiovasc. Surg..

[5] Klemperer P., Coleman B.R. (1992). Primary Neoplasms of the Pleura. A Report of Five Cases. Am. J. Ind. Med..

[6] Stout A.P., Himadi G.M. (1951). Solitary (Localized) Mesothelioma of the Pleura. Ann. Surg..

[7] Attanoos R.L., Pugh M.R. (2018). The Diagnosis of Pleural Tumors Other Than Mesothelioma. Arch. Pathol. Lab. Med..

[8] Yonli D.S., Chakroun M., Mokadem S., Saadi A., Rammeh S., Chebil M. (2019). Adrenal Solitary Fibrous Tumor: A Case Report. Urol. Case Rep..

[9] Ronchi A., Cozzolino I., Zito M.F., Accardo M., Montella M., Panarese I., Roccuzzo G., Toni G., Franco R., De Chiara A. (2018). Extrapleural Solitary Fibrous Tumor: A Distinct Entity From Pleural Solitary Fibrous Tumor. An Update on Clinical, Molecular and Diagnostic Features. Ann. Diagn. Pathol..

[10] Song Z., Yang F., Zhang Y., Fan P., Liu G., Li C., Ding W., Zhang Y., Xu X., Ye Y. (2018). Surgical Therapy and Next-Generation Sequencing-Based Genetic Alteration Analysis of Malignant Solitary Fibrous Tumor of the Pleura. Onco Targets Ther..

[11] England D.M., Hochholzer L., McCarthy M.J. (1989). Localized Benign and Malignant Fibrous Tumors of the Pleura. A Clinicopathologic Review of 223 Cases. Am. J. Surg. Pathol..

[12] Mendez-Sanchez H., Mendez-Vivas W., Vargas-Mendoza G.K., Vazquez-Lopez S., Williams-Jacquez A.D., Cortes-Telles A. (2019). Solitary Fibrous Tumors of the Pleura: A Clinical-Pathological Characterization Emphasizing Changes in Lung Function. Adv. Respir. Med..

[13] Briselli M., Mark E.J., Dickersin G.R. (1981). Solitary Fibrous Tumors of the Pleura: Eight New Cases and Review of 360 Cases in the Literature. Cancer.

[14] Jha V., Gil J., Teirstein A.S. (2005). Familial Solitary Fibrous Tumor of the Pleura: A Case Report. Chest.

[15] Cardillo G., Facciolo F., Cavazzana A.O., Capece G., Gasparri R., Martelli M. (2000). Localized (Solitary) Fibrous Tumors of the Pleura: An Analysis of 55 Patients. Ann. Thorac. Surg..

[16] Urbina-Lima A.D., Roman-Martin A.A., Crespo-Santos A., Martinez-Rodriguez A., Cienfuegos-Belmonte I.R., Olmo-Ruiz M., Esteban-Artiaga R., Molina-Suarez J.L. (2019). Solitary Fibrous Tumor of the Urinary Bladder Associated with Hypoglycemia: An Unusual Case of Doege-Potter Syndrome. Urol. Int..

[17] Prado F., Dos Ramos J.P., Larranaga N., Espil G., Kozima S. (2018). Solitary Fibrous Tumor and Doege-Potter Syndrome. Medicina (B Aires).

[18] Yanik F., Karamustafaoglu Y.A., Yoruk Y. (2019). Surgical Outcomes and Clinical Courses of Solitary Fibrous Tumors of Pleura. Niger. J. Clin. Pract..

[19] Tapias L.F., Mercier O., Ghigna M.R., Lahon B., Lee H., Mathisen D.J., Dartevelle P., Lanuti M. (2015). Validation of a Scoring System to Predict Recurrence of Resected Solitary Fibrous Tumors of the Pleura. Chest.

[20] Lahon B., Mercier O., Fadel E., Ghigna M.R., Petkova B., Mussot S., Fabre D., Le Chevalier T., Dartevelle P. (2012). Solitary Fibrous Tumor of the Pleura: Outcomes of 157 Complete Resections in a Single Center. Ann. Thorac. Surg..

[21] de Perrot M., Fischer S., Brundler M.A., Sekine Y., Keshavjee S. (2002). Solitary Fibrous Tumors of the Pleura. Ann. Thorac. Surg..

[22] Ventura L., Gnetti L., Braggio C., Carbognati P., Rusca M., Silini E.M., Ampolini L. (2017). Solitary Fibrous Tumor of the Pleura Associated with Severe Hypoglicemia: The Doege-Potter syndrome. J. Thorac. Oncol..

[23] Galateau-Salle F., Churg A., Roggli V., Travis W.D. (2016). The 2015 World Health Organization Classification of Tumors of the Pleura: Advances since the 2004 Classification. J. Thorac. Oncol..

[24] Huang S.C., Huang H.Y. (2019). Solitary Fibrous Tumor: An Evolving and Unifying Entity with Unsettled Issues. Histol. Histopathol..

[25] Ghanim B., Hess S., Bertoglio P., Celik A., Bas A., Oberndorfer F., Melfi F., Mussi A., Klepetko W., Pirker C. (2017). Intrathoracic Solitary Fibrous Tumor—An International Multicenter Study on Clinical Outcome and Novel Circulating Biomarkers. Sci. Rep..

[26] Cao Y.Y., Fan N., Xing F., Xu L.Y., Qu Y.J., Liao M.Y. (2015). Computed Tomography-Guided Cutting Needle Pleural Biopsy: Accuracy and Complications. Exp. Ther. Med..

[27] Mavarez J.D.A., Montes M.A.V., Seoane M.R.R., Shao M.L. (2019). Hypoglycemia as an Atypical Presentation of a Pleural Tumor. Arch. Bronconeumol..

[28] Karki A., Yang J., Chauhan S. (2018). Paraneoplastic Syndrome Associate with Solitary Fibrous Tumor of Pleura. Lung India.

[29] Meng W., Zhu H.H., Li H., Wang G., Wei D., Feng X. (2014). Solitary Fibrous Tumors of the Pleura With Doege-Potter Syndrome: A Case Report and Three-Decade Review of the Literature. BMC Res. Notes.

[30] Gomez F.D., Robin L., Jakubowicz D., Sillou S., Lab J.P., Balian C. (2019). Solitary Fibrous Tumor of the Retroperitoneum With Urinary Symptoms Revealing a Doege-Potter’s Syndrome. Prog. Urol..

[31] Tapias L.F., Lanuti M. (2015). Solitary fibrous tumors of the pleura: Review of literature with up-to-date observations. Lung Cancer Manag..

[32] Jannin A., Espiard S., Benomar K., Do C.C., Mycinski B., Porte H., D’Herbomez M., Penel N., Vantyghem M.C. (2019). Non-Islet-Cell Tumour Hypoglycaemia (NICTH): About a Series of 6 Cases. Ann. Endocrinol. (Paris).

[33] Aridi T., Tawil A., Hashem M., Khoury J., Raad R.A., Youssef P. (2019). Unique Presentation and Management Approach of Pleural Solitary Fibrous Tumor. Case Rep. Surg..

[34] Wada Y., Okano K., Ando Y., Uemura J., Suto H., Asano E., Kishino T., Oshima M., Kumamoto K., Usuki H. (2019). A Solitary Fibrous Tumor in the Pelvic Cavity of a Patient with Doege-Potter Syndrome: A Case Report. Surg. Case Rep..

[35] Kim D.W., Na K.J., Yun J.S., Song S.Y. (2017). Doege-Potter Syndrome: A Report of a Histologically Benign but Clinically Malignant Case. J. Cardiothorac. Surg..

[36] Han G., Zhang Z., Shen X., Wang K., Zhao Y., He J., Gao Y., Shan X., Xin G., Li C. (2017). Doege-Potter Syndrome: A Review of the Literature Including a New Case Report. Medicine (Baltimore).

[37] Villemain A., Menard O., Mandry D., Siat J., Vignaud J.M., Martinet Y., Tiotiu A. (2017). Paraneoplastic Hypoglycemia: The Hopes of Pathophysiological Documentation. Rev. Pneumol. Clin..

[38] Kuhn-Velten U., Hohmann C., Strauss T., Heizmann O., Kloppel G. (2018). Solitary Fibrous Tumor: A Rare Cause of Recurrent Severe Hypoglycemia. Dtsch. Med. Wochenschr..

[39] Rena O., Filosso P.L., Papalia E., Molinatti M., Di Marzio P., Maggi G., Oliaro A. (2001). Solitary Fibrous Tumour of the Pleura: Surgical Treatment. Eur. J. Cardiothorac. Surg..

[40] Pirvu A., Angelescu D., Savu C. (2016). Localized Fibrous Tumor of the Pleura an Unusual Cause of Severe Hypoglycaemia. Case Report. Rev. Med. Chir. Soc. Med. Nat. Iasi.

[41] Bailly C., Bichali A.M., Douane F., Ansquer C., Drui D. (2018). Metastatic Solitary Fibrous Tumor with Doege-Potter Syndrome: Hypoglycemia Treated by 90Y Radioembolization. Clin. Nucl. Med..

[42] Ogunsakin A.A., Hilsenbeck H.L., Portnoy D.C., Nyenwe E.A. (2018). Recurrent Severe Hypoinsulinemic Hypoglycemia Responsive to Temozolomide and Bevacizumab in a Patient With Doege-Potter Syndrome. Am. J. Med. Sci..

[43] Sugimoto K., Tokitou R., Kadosaki M., Takeuchi M. (2019). Intraoperative Glycemic Control Using an Artificial Endocrine Pancreas in a Patient with a Recurrent Pleural Solitary Fibrous Tumor Producing Insulin-Like Growth Factor 2: A Case Report. JA Clin. Rep..

[44] Bossart S., Rammlmair A., Haneke E. (2019). Reversible Schamroth Sign after Pleural Tumor Resection. Skin Appendage Disord..

[45] Galie N., Vasile R., Savu C., Petreanu C., Grigorie V., Tabacu E. (2010). Superior Vena Cava Syndrome—Surgical Solution—Case Report. Chirurgia (Bucur.).

[46] Shiono S., Abiko M., Tamura G., Sato T. (2009). Malignant Solitary Fibrous Tumor with Superior Vena Cava Syndrome. Gen. Thorac. Cardiovasc. Surg..

[47] You X., Sun X., Yang C., Fang Y. (2017). CT Diagnosis and Differentiation of Benign and Malignant Varieties of Solitary Fibrous Tumor of the Pleura. Medicine (Baltimore).

[48] Cardinale L., Dalpiaz G., Pulzato I., Ardissone F. (2018). Computed Tomography of Solitary Fibrous Tumor of the Pleura Abutting the Mediastinum: A Diagnostic Challenge. Lung India.

[49] Aluja J.F., Gutierrez F., Bhalla S. (2018). Pleural Tumours and Tumour-Like Lesions. Clin. Radiol..

[50] Abu A.W. (2012). Solitary Fibrous Tumours of the Pleura. Eur. J. Cardiothorac. Surg..

[51] Gupta A., Souza C.A., Sekhon H.S., Gomes M.M., Hare S.S., Agarwal P.P., Kanne J.P., Seely J.M. (2017). Solitary Fibrous Tumour of Pleura: CT Differentiation of Benign and Malignant Types. Clin. Radiol..

